# Supplementary material for: Associations of temperature and precipitation with malaria in children under 5: A multi-country study in Sub-Saharan Africa
Source: PLoS One. 2025 Oct 27;20(10):e0335031. doi: 10.1371/journal.pone.0335031 (PMC12558544; doi:10.1371/journal.pone.0335031)
Supplement: Attachment — Submitted filename: Comments.pdf [file pone.0335031.s001.pdf]

Comments

1. **Title:**

The manuscript title, main objective, and conclusions are consistent.

2. **Method:**

The data sources are valid (DHS, ERRA-5). However, details must be detailed in the methodology to better judge the validity of the results and facilitate the reproducibility of analysis:

a. Reference year of the DHS of the countries;

b. inclusion criteria used by the DHS to include the case of malaria. Did these criteria differ according to the countries?

c. Precision of the spatial scale (cluster) of meteorological data collection in the countries;

d. note on the method of calculating certain variables such as temperature density.

3. Discussions

It is recognized in the literature that the use of insecticide-treated mosquito nets has a significant impact on malaria transmission. It would be worth discussing the possible limitations leading to the insignificance of this intervention in this study.

4. Conclusion :

The conclusion is consistent with the title and the objective.

NB: I didn’t have access to the database to answer certain questions noted above.
