# Supplementary material for: Associations of temperature and precipitation with malaria in children under 5: A multi-country study in Sub-Saharan Africa
Source: PLoS One. 2025 Oct 27;20(10):e0335031. doi: 10.1371/journal.pone.0335031 (PMC12558544; doi:10.1371/journal.pone.0335031)
Supplement: Attachment — Submitted filename: Response to reviewers 130825.docx [file pone.0335031.s002.docx]

FOR REVIEWER 1

RESPONSE MATRIX

| COMMENT | RESPONSE |
| --- | --- |
| Ensure consistency in decimal formatting throughout the abstract and manuscript (e.g., using either one or two decimal places uniformly). | Thank you for this feedback. For the estimates and values for confidence intervals, we used 3 decimal places, and one decimal place for percentages, as some of the results such as p-value <0.001, reporting it as <0.01 could be misleading. |
| Line 110-113: "Although numerous studies have investigated the overall health impacts of climate change, the specific interplay between shifting climatic conditions and malaria transmission dynamics in vulnerable populations remains underexplored." To strengthen this statement, you should cite relevant studies that examine both the general health effects of climate change and those that focus on malaria transmission dynamics | Citations added. |
| The study design should be clearly stated. The Demographic and Health Survey (DHS) follows a two-stage stratified cluster sampling design, and this should be properly described in the Study Design and Population section.  The authors should specify how sample weights were implemented in the data analysis section. Applying sample weights is crucial to ensure that the findings are representative of the target population. | A clear study design was added, and sampling procedure was clearly stated.  Sampling weight was clearly specified. |
| Line 340-42: Remove this "These findings reinforce the consistently high malaria prevalence in SSA and highlight the need for continued surveillance and intervention efforts to reduce the disease burden among children under five | The pre-mentioned lines were included to preamble the discussion part. We thought putting these statements would add value to the discussion part. |
| Line 348-50: Remove this" These results highlight the multifaceted nature of malaria transmission and the need for integrated control strategies that incorporate both environmental and socio-economic determinants." | The statements were removed. |
| A Policy Recommendations' section should be added before the Conclusion to provide actionable insights based on the study’s key findings. This section should highlight policy implications and suggest evidence-based interventions | Policy recommendations added after the conclusion. |

For Reviewer 3

| Comment | Response |
| --- | --- |
| Add reference year of the used country respective DHS. | Reference years were added in Table 1 under the **DHS country Data (Year)** |
| inclusion criteria used by the DHS to include the case of malaria. Did these criteria differ according to the countries? | Inclusion and exclusion criteria were added.  The inclusion and exclusion criteria did not differ according to the country. |
| . Precision of the spatial scale (cluster) of meteorological data collection in the countries; | Thank you for your valuable comment. In this study, we used ERA-5 reanalysis data, which provides gridded meteorological variables at approximately 0.25° × 0.25° (~28 km × 28 km) spatial resolution. To integrate these data with DHS cluster locations, we employed spatial interpolation (or spatial averaging) to assign ERA5-derived climate variables (temperature and precipitation) to each DHS cluster based on its GPS coordinates.  However, we have updated our manuscript by adding the following information to clarify our approach:  *“We used zonal averaging (area-weighted averaging) to assign ERA-5 derived climate variables to DHS clusters. Specifically, for each cluster, we extracted and averaged values from all ERA5 grid cells within a defined buffer zone (10 km radius for each cluster), ensuring a more representative estimate of local climate conditions. We used this approach is appropriate for handling the spatial displacement of DHS cluster coordinates and provides more robust estimates”.* |
| note on the method of calculating certain variables such as temperature density | A note was taken care of. |
| It is recognized in the literature that the use of insecticide-treated mosquito nets has a significant impact on malaria transmission. It would be worth discussing the possible limitations leading to the insignificance of this intervention in this study | One possible explanation is the reliance on self-reported data for ITN use, which may be subject to recall or social desirability bias, leading to misclassification. Additionally, our analysis was cross-sectional and may not fully capture seasonal variations in both ITN use and malaria transmission. It is also possible that high community-level ITN coverage in some areas reduced measurable differences between users and non-users, or that factors such as net age, physical condition, and proper use were not adequately captured in the dataset. Finally, variations in vector resistance to insecticides across the study settings could attenuate the effectiveness of ITNs. We have now included these points in the discussion to acknowledge these limitations and provide context for the observed results. |

Reviewer 2

| Comment | Response |
| --- | --- |
| Line 214: The use of a p-value threshold (0.2) alone for variable selection is not robust. Please consider using AIC/BIC or stepwise regression for a more defensible selection process. | Thank you for the input. We acknowledge that AIC and BIC are commonly applied for model selection when comparing multiple competing models to determine the best fit. However, in our study, a p-value threshold of less than 0.2 was employed specifically for variable selection—transferring variables from the univariable model to the multivariable model. This approach was not intended for overall model selection but rather served as a criterion for identifying relevant predictors for multivariable analysis. |
| Lines 227–236: Clarify whether the “Overall” prevalence in Figure 1 is weighted across countries. | Thank you regarding this input.  All analyses performed in the study were weighted as have been highlighted in the methodology section. |
| Lines 244–253: Figures 2 and 3 would benefit from clearer x-axis labels and indication of temperature or precipitation range across clusters. Consider labeling country groups directly in density curves. | Thank you again for this input.  **Figures 2 and 3** were revised to take into account the country groupings. |
| Lines 313–317: Since a non-linear temperature effect is observed, consider using spline regression or a GAM to model the dose–response more flexibly, and illustrate it graphically. Discussion (Lines 331–462) | Thank you for the input.  The inclusion of a squared temperature term in the model effectively captured the nonlinear relationship between temperature and malaria, aiding in model linearization. Given that this transformation yielded satisfactory model performance, we did not find it necessary to pursue more complex modeling techniques such as spline regression and GAM. |
| 17.Table 2 : Clearly specify reference categories in footnotes (e.g., “Secondary education vs. no education”). | Thank you for the input.  All reference categories presented in Table 2 of the originally submitted manuscript were explicitly identified by assigning the code “1” to the respective reference group in each categorical variable. The footnote was added to clearly define the reference group under Table 2. |
| 16.Figures 1–5: Improve figure legends, label axes, and standardize the color scales used in heat maps. A clear legend indicating levels of malaria risk would help. | Thank you for the input.  We considered the option you suggested, but it could not be implemented in that form. Since temperature and precipitation were treated as continuous variables in the heat maps, deriving discrete malaria risk levels proved methodologically problematic. Therefore, we opted to retain the original figure format.  In the heat maps, the red coloration reflects the density of reported malaria cases. Deep red areas indicate high concentrations of cases at specific temperature ranges, while lighter red bands correspond to lower case counts. This visual gradient preserves the continuous nature of the underlying climatic variables and aligns with the original analytical approach. |
| 15.Lines 497–500: The recommendation for future research could be better structured. Suggest explicitly that longitudinal and cohort studies are needed to establish causality and clarify lagged climate–malaria effects. Others | Thank you for the suggestion.  The explicit recommendation based on longitudinal study for establishing causality have now been added. |
| Lines 348–368: The mechanisms linking temperature to mosquito behavior and malaria transmission are over-detailed and speculative in parts (e.g., “light clothing increases bite exposure”). Please ensure all statements are backed by references. | Thank you for the input.  Redundant statements have been removed and citations have been added where necessary. |
| Lines 142–150: Clarify that “hml35” refers to positive malaria rapid diagnostic test results. | Thank you for the input.  Hml35 was re-defined as “positive malaria rapid diagnostic test results” as suggested. |
| . Lines 20–46: The abstract contains awkward phrasing. For example, “a 1.77-fold in risks” should be revised to “a 1.77-fold increase in risk,” and “0.99-fold in risks” to “a 1% decrease in risk.” The overall writing should be polished for conciseness and clarity. Introduction (Lines 47–120 | Thank you. The entire results of the Abstract has been rewritten for clarity. |
| . Reinforce the definition of the exposure window (12 months prior to survey) for temperature and precipitation to strengthen the logic of temporal association | Thank you.  Reinforcement statement was added. |
| Lines 109–117: Please highlight the novelty of your study. For example, emphasize that this is one of the few multi-countries SSA studies combining ERA-5 climate data with DHS microdata to examine under-five malaria risk. | Thank you for the suggestion.  Statements were added to highlight the novelty of the study. |
| . Lines 1–2: The current title is overly long. Consider simplifying to: “Climatic Factors and Malaria Risk among Under-Five Children in Sub-Saharan Africa: A Multi-Country Analysis.” | The title was revised. |
