# Supplementary material for: Associations of temperature and precipitation with malaria in children under 5: A multi-country study in Sub-Saharan Africa
Source: PLoS One. 2025 Oct 27;20(10):e0335031. doi: 10.1371/journal.pone.0335031 (PMC12558544; doi:10.1371/journal.pone.0335031)
Supplement: Attachment — Submitted filename: Response to reviewers_02October2025.docx [file pone.0335031.s003.docx]

**Response to reviewers**

| SN | Comment | Response | Line # |
| --- | --- | --- | --- |
| 1 | The title currently reads “Children Under5”. Please correct the spacing to “Children Under 5. | Thank you.  The spacing was corrected. | 2 |
| 2 | The word “drivers” (Line 1) suggests causality, which is not appropriate for a cross-sectional study. A more neutral term such as “associations” or “relationships” would be preferable. | Thank you for the comment.  The word “drivers” was removed, and the title was revised on the reviewer’s suggestion. | 1 |
| 3 | Lines 20–45: The abstract remains relatively long. Consider condensing it to approximately 300 words according to the requirements of the journal, focusing on the study objective, main findings, and implications rather than detailed statistics | Thank you for the comment.  The abstract has been revised to 263 words. | 36-53 |
| 4 | Lines 227–236: The explanation of weighting remains brief. Please include the DHS weighting formula in the main text (e.g., weight = v005/1,000,000) and reference DHS methodological documentation, not only supplementary notes. | Thank you for the comment.  The weighting formula was included in the previously submitted manuscript. | 242 |
| 5 | Lines 259–263: The narrative duplicates what is shown in Figure 1. A more concise statement is recommended, such as: “Prevalence varied considerably, from below 10% in Tanzania to over 30% in Nigeria and Burundi (Figure 1). | Thank you for your comment. The narrative reports the computed prevalence for each of the six countries, while Figure 1 illustrates the overall prevalence range across the sample. We consider it important to keep precise country-specific estimates rather than relying only on the broader range shown in the figure. Nevertheless, we have revised the text to be more concise in line with your suggestion |  |
| 6 | Some passages are repetitive, for example, Lines 379–384 and 388–397 both emphasize the importance of temperature for malaria transmission. Consider merging and streamlining these sentences to improve clarity. | Thank you for the comment. The sentences have been restructured to enhance clarity and eliminate redundancy. | 324 |
| 7 | The policy recommendations starting at Line 562 appear disconnected from the conclusion. I suggest integrating them into the final Discussion paragraph so they flow naturally into the Conclusion | Appreciated for the comment. We revised the recommendations to align with the findings in the discussion and conclusion as suggested by the reviewer. | 578-600 |
| 8 | Line 246: The use of p<0.2 as a criterion for variable inclusion lacks sufficient justification. Consider adding a sensitivity analysis (e.g., with p<0.1) to show robustness, or cite relevant methodological literature. | Citation supporting the use of the criterion was added | 258 &  259 |
|  |  |  |  |
